# Innovation in Acute Ischemic Stroke Patients over 80 y/o—A Retrospective Monocentric Study on Mechanical Thrombectomy of Consecutive Patients: Is Age an Adequate Selection Criterion?

**DOI:** 10.3390/jcm12113688

**Published:** 2023-05-26

**Authors:** Massimiliano Cernigliaro, Carmelo Stanca, Andrea Galbiati, Marco Spinetta, Carolina Coda, Davide Negroni, Domenico Laganà, Roberto Minici, Chiara Airoldi, Alessandro Carriero, Giuseppe Guzzardi

**Affiliations:** 1 Radiodiagnostica ed Interventistica, Azienda Ospedaliero Universitaria Maggiore della Carità, 28100 Novara, Italy; 2 Radiology Unit, Dulbecco University Hospital, 88100 Catanzaro, Italy

**Keywords:** stroke, elderly, innovation, brain, mechanical thrombectomy, direct aspiration, stent retriever, decision-making, age, vessel, atherosclerosis, hypertension, smoke, angiography, solumbra

## Abstract

Background: Although it is clear that stroke is a time-dependent and age-associated disease, we still need more evidence regarding the efficacy and outcomes in elderly patients who were excluded from the first trials of mechanical thrombectomy. The aim of this study is to highlight patient characteristics, the timing of medical attention and therapy, successful recanalization, and functional outcomes in patients over 80 y/o who underwent mechanical thrombectomy at the Ospedale Maggiore della Carità di Novara (Hub) since endovascular stroke treatment was first started here. Methods: all 122 consecutive patients over 80 y/o at admission who underwent mechanical thrombectomy between 2017 and 2022 at our Hub center were retrospectively included in our database. A good functional outcome in these elderly patients was considered as the 90 days modified Rankin Scale (mRS) ≤ 3 and/or a decrease in functional status as ∆mRS ≤ 1 in order to interpret the results for patients with intact intellect and basal mRS > 3. Successful recanalization as a score of TICI ≥ 2b (Thrombolysis in Cerebral Infarction) was analyzed as a secondary outcome. Results: Good functional outcome (mRS ≤ 3 and/or ∆mRS ≤ 1) was observed in 45.90% (56/122). The rate of successful recanalization (TICI ≥ 2b) was 65.57% (80/122). Conclusion: Our data confirm that a good outcome in the elderly age group has a correlation with age; being younger, with a milder NIHSS (National Institutes of Health Stroke Scale) at the onset and with a lower pre-morbid mRS is statistically associated with a better outcome. However, age should not be a criterion to exclude older patients from mechanical thrombectomy. Decision-making should take into consideration the pre-morbid mRS and the severity of the stroke on the NIHSS scale, especially in the age group over 85 y/o.

## 1. Background

Stroke is the second leading cause of death worldwide and the main cause of long-term neurological disability in adults [1]. Due to the ageing population, the absolute number of strokes is expected to increase dramatically in the coming years in Europe; by 2025, 1.5 million Europeans will suffer a stroke each year. Beyond vital prognosis, stroke patients are also at an increased risk of poor outcomes within the first year of the event, including rehospitalization, event recurrence, dementia, mild cognitive disorder, depression, and fatigue, all affecting the health-related quality of life [2]. This represents a serious issue, especially in Italy, where the ratio of elderly people is always on the rise, with public healthcare being free for everybody and causing high hospitalization and rehabilitation costs. It is known that in high-income countries, about a third of all strokes occur in this age bracket [3].

Endovascular therapy has now been consolidated as the mainstay therapy for large vessel occlusion, together with systemic fibrinolysis [4], however, even though a stroke is an age-related illness connected with the cardiovascular risk profile, the first multicenter trials predominantly investigated outcomes of mechanical thrombectomy in patients < 80 y/o [5]. Data from bigger trials, such as HERMES, demonstrated good outcomes in the elderly group, however, these patients were underrepresented [6]; smaller trials indicate that endovascular therapy associated with fibrinolysis in the elderly group could achieve a good outcome as well, with mixed results [7,8]. Data on this matter are valuable in order to reach a strong consensus on which is the best therapy for this age group, especially to find if there is an upper age limit that contraindicates mechanical thrombectomy, as well as different timings or factors that might prevent patients from achieving a good outcome.

## 2. Methods

### 2.1. Population

Since 2017, the Ospedale Maggiore della Carità di Novara has implemented endovascular therapy for stroke treatment as a hub center for a large territory, including 3 spoke hospitals. In this study, we retrospectively included all patients between June 2017 and November 2022 with an age of ≥80 y/o at admission and intracranial vessel occlusion who underwent mechanical thrombectomy. The only exclusion criterion was an age of <80 y/o. Our general inclusion criteria for treatment included patients presenting in the correct time window at <6 h from the onset of symptoms or when a reasonable ratio of salvageable penumbra/ischemic core < 50% was detected on CT perfusion imaging up to 24 h from the onset. Patients with intact intellectual functioning, regardless of their pre-morbid mRS (modified Rankin Scale), were accepted for treatment and followed for 3 months after admission; therefore, pre-morbid mRS was not an exclusion criterion. Regarding this study, the following clinical data were collected: main diagnosis with etiology classified using TOAST (Trial of Org 10172 Acute Stroke Treatment) classification, symptoms at admission and discharge evaluated based on NIHSS (National Institute of Health Stroke Scale) scale, pre- and post-morbid mRS, thrombolytic therapy, time to CT, time to fibrinolysis and to the groin, procedural time, main mechanical technique used (direct aspiration, stent retriever, both, or rescue treatment when either failed), number of passages and recanalization rate based on TICI scale (Thrombolysis in Cerebral Infarction), hemorrhage at CT based on ECASS (European Cooperative Acute Stroke Study) classification. Comorbidities, such as hypertension, hypercholesterolemia, diabetes, atrial fibrillation, smoking, and coronary artery disease, were collected. We also classified the onset time in order to see if there were any differences in our practice during the day or night.

**Figure 1:**
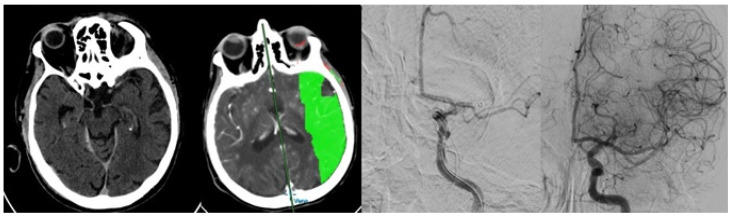
An 82 y/o with left m2 occlusion undergoing recanalization with single pass direct aspiration.

In the operating room, we usually obtain the right femoral access and navigate using a triaxial catheter system in order to reach the site of occlusion. The choice of the mechanical technique used is left to the interventional radiologist, however, it usually involves two attempts with either direct aspiration [9,10,11] or a stent retriever combined with direct aspiration (“Solumbra”) [12]. When it is impossible to reach the occlusion with the aspiration catheter, only a stent retriever technique is used [13]. The third, and all further attempts, usually involve a different (“rescue”) method (“rescue”), either by combining a stent for direct aspiration only or by changing the type of stent [14,15] or the aspiration catheter used [16,17] (Figure 1).

### 2.2. Statistical Analysis

Descriptive statistics were reported for the whole sample using absolute and percentage frequencies for categorical variables, while means and standard deviations, or medians and interquartile ranges, were used for numerical variables, as appropriate.

To evaluate the success of the treatment, functional mRS and procedural outcomes based on the TICI scale were considered. Particularly, a success was considered if the pre-morbid and 3-month mRS difference (∆mRS) was less than 1 point, or the mRS at 3 months was 3 or less (primary outcome), and the TICI was >2a (2b, 2c, or 3). The association between outcomes and covariates was evaluated using the chi-square test, Fisher and *t*-test, or non-parametric alternatives, as appropriate. Then, univariate and multivariable logistic models were performed, including only the covariates that maintained the statistical significance.

Finally, a sensitivity analysis was conducted to make a comparison with other studies in the literature, considering that the only main outcome was a post-morbid mRS of ≤3.

The statistical threshold was set to 0.05 (two-tailed), and the analysis was conducted using the SAS 9.4.

### 2.3. Ethics Approval

This is a retrospective study conducted in a single Italian center. Patients’ informed consent for the procedure was acquired when possible, according to the hospital’s protocol. Ethical review and approval were not required in accordance with the national guidelines and institutional requirements.

## 3. Results

We included 122 patients who were >80 y/o, with a mean of 84 y/o, 43 (35.25%) being over 85, and 14 (11.48%) over 90 years old. (Table 1)

Our sample included more female (85; 69.67%) than male patients (37; 30.33%). The ischemic stroke involved the posterior circulation in 8 patients (6.56%), while the anterior circulation in 114 (93.44%), with 54 (47.3%) involving the right anterior circulation, and 61 (52.7%) involving the left anterior circulation. Regarding the etiology, 62 (51.67%) were considered of cardioembolic origin, with large atherosclerosis as the main factor in 38 (31.67%) and stroke being due to other causes in 20 (16.67%).

Out of all patients, 45 (36.89%) were sent by spoke hospitals, while the rest (77; 63.31%) arrived at the hub center in Novara directly. Around 108 patients had a set onset time of symptoms, while 14 (11.48%) were wake-up stroke, with a time of onset calculated at the midpoint between the sleep onset (or last known to be normal) and the time of waking up. Fibrinolysis was performed in 65 patients (54.62%), while others presented contraindications, such as anticoagulation therapy, recent major surgeries, etc. Approximately eight (6.56%) patients had no comorbidities, 31 (25.41%) had more than one comorbidity, and 83 (68.03) had two or more comorbidities.

The mean pre-morbid mRS was 1.4 in our study population, with a severe median NIHSS at onset (18). The times to medical attention and therapy were long, with a mean of 121.74 min from symptom onset to CT, a mean of 172.25 min to fibrinolysis, and a mean of 262.42 min to groin femoral punctures. The mean procedural time was around 56 min. Around 34 (27.87%) patients were admitted and treated during the night “on-call” shift (between 20:00 and 08:00).

Intracranial hemorrhage occurred in 32.79% (40/122) of patients. In-hospital mortality was 16% (20/122).

The first pass technique was a direct aspiration in 77 (64.17%), stent retriever in 5 (4.17%), and direct aspiration combined with stent retriever (“Solumbra technique”) in 38 (31.67%). The end result was achieved with one pass in 69 (56.5%), while two or more attempts were needed in 53 (43.44%). A “rescue” technique was needed in 20 (16.39%) when the first pass technique failed (combining either a stent retriever or direct aspiration).

The univariate analysis for predictors of a good outcome at 90 days after endovascular thrombectomy found statistically better functional outcomes associated with age (*p* = 0.005) in patients presenting with a milder stroke on the NIHSS scale (*p* = 0.001) (Table 2). Unfortunately, a bad clinical outcome was found to be statistically associated with patients coming from a spoke hospital (*p* = 0.012) and was associated with hemorrhage (*p* = 0.001) and age over 85 y/o (*p* = 0.010). Although smoke (*p* = 0.004) and hypercholesterolemia (*p* = 0.029) were found to be predictors of good functional outcomes in our population, we think this could have been due to a population bias in comorbid patients, as they were not statistically associated with a higher recanalization rate and were not statistically significant when grouped together in the multivariate analysis. Moreover, there were no differences in results in patients treated during nighttime or daytime.

A multivariable logistic model was performed, including the covariates that were statistically significant in the univariate model. A milder NIHSS at admission was found to be an independent predictor of good functional outcomes in multivariable analysis (Table 3).

Successful recanalization (Table 4) was associated with age (*p* = 0.0002).

Age above 85 y/o was also associated with unsuccessful recanalization (*p* = 0.0011). Combining a “rescue technique” (either stent retriever or direct aspiration) with our first pass technique did not achieve a better recanalization rate and was associated with unsuccessful recanalization (*p* = 0.0342). Additionally, longer procedural times were associated with unsuccessful recanalization, however, this could be due to the fact that when recanalization was not achieved, the radiologist made all the necessary efforts and used all the time needed to try to achieve the best result for the patient.

We also analyzed data considering only mRS ≤ 3 as the main outcome without adding ∆mRS ≤ 1 to be able to make a comparison with other studies in the literature (Table 5). Our main results do not really change, except for good outcomes associated with a lower pre-morbid mRS (p0.01).

## 4. Discussion

Successful recanalization in the literature is reported from 96% [18] to 78.5% [19], as well as 54.2% [20] in patients aged 80 years or older. In our cohort, we achieved a 65.57% successful recanalization rate, which is in line with the literature, however, it can still be improved. A reason for the lower success rate could be explained by the fact that a prolonged time from onset to reperfusion could cause clot organization and make it harder to retrieve, although this is just speculative.

A meta-analysis of five trials of mechanical thrombectomy reported a 90-day mortality with a 15.3% death rate and a median age of 68 years [5], while other studies reported a much higher rate of 40.9% in the >80 y/o group [19]. The death rate in our cohort is around 16%, which is in line with some reported articles and thanks to the work of our Stroke and Intensive Care Unit for these comorbid patients that could die not only from hemorrhage or stroke-related complications but also from pneumonia, heart failure, infections, or other age-related complications related to hospitalization.

The time from onset to CT (121.74 min), to fibrinolysis (172.25 min), and to groin femoral access (262.42 min) are very long in our cohort, as previously mentioned, and they show that there is room for improvement. Several organizational factors could determine the long time to access mechanical reperfusion therapies [21]. We all embrace the saying that “time is brain” [22]^,^ and this surely affects the success and outcomes of mechanical thrombectomy, which, even when performed correctly, might achieve worse results due to prolonged brain ischemia. Many of the methods used to shorten the time to reperfusion are discussed in the literature, ranging from the pre-hospital patient and emergency services education protocols with pre-notification to the receiving hospital, to dedicated in-hospital pathways. Out of all these, we are now discussing the possibility of skipping the double in-hospital time bureaucracy for patients initially admitted in a spoke-hospital by accepting them directly to an angiography suite in our hub center in Novara instead of passing through our emergency room again.

Age, milder NIHSS at onset, and a lower mRS were associated with better outcomes, which was in line with previous stroke studies considering other age groups. Obviously, younger patients with a less severe stroke, who were self-sufficient at home, achieved better results; however, considering the whole cohort, considering the fact that we did not set an age limit to access treatment, success in recanalization and outcomes was achieved, improving patients’ overall functional independence and possibly reducing caregivers’ burden.

## 5. Limitations

The main limitation of this study is the retrospective nature of this work, however, all consecutive patients over 80 y/o were included in order to represent the real-life experience in our center. All cases were from a single institution with standard management and procedural protocols to help minimize procedural variability, however, this limits the cohort of patients (n = 122), which is already quite large for being a monocentric study. However, in order to achieve a general consensus and eliminate biases related to internal protocols, multicentric studies are necessary.

## 6. Conclusions

Our data confirm that a good outcome in the elderly age group has a correlation with age; being younger, with a milder NIHSS at the onset and lower pre-morbid mRS is statistically associated with a better outcome. Patients over 85 y/o had worse functional outcomes, with lower recanalization rates. Overall, good functional outcomes were observed in 45.90%, with a 16% mortality, which is acceptable and comparable to other studies.

Age should not be a criterion to exclude older patients from mechanical thrombectomy, although in clinical practice, decision-making should take into consideration pre-morbid mRS and the severity of stroke on the NIHSS scale, especially in the age group over 85 y/o.

## Figures and Tables

**Table 1:** Study population characteristics, such as clinical data, site of occlusion, technique used, and timing to therapies.

Patients Characteristics		n = 122
***Sex***	Male	37 (30.33%)
	Female	85 (69.67%)
***Age, years***		
*Mean (SD)*		84.3 (3.60)
*Median [q1-Q3]*		83 [82; 86]
***Cerebral Circulation***	Anterior	114 (93.44%)
	Posterior	8 (6.56%)
***Anterior Circulation. Side***	Right	55 (47.41)
	Left	61 (52.59)
***Spoke***		45 (36.89%)
***Wake up Stroke***		14 (11.48%)
***Fibrinolysis***		65 (54.62%)
***Device***	Direct Aspiration	77 (64.17)
	Stent retriever	5 (4.17)
	“Solumbra”	38 (31.67)
***N_attempts***	1	69 (56.5%)
	2+	53 (43.44%)
***Rescue***		20 (16.39%)
*Median [q1-Q3]*		1 [1; 2]
***Hemorrhage***	No	82 (67.21%)
	Yes	40 (32.79%)
	H1	9 (7.38%)
	H2	10 (8.2%)
	PH1	9 (7.38%)
	PH2	12 (9.84%)
***Comorbidities***		
*Hypertension*		82 (67.21%)
*Smoke*		20 (16.39%)
*Coronary Artery Disease*		38 (31.15%)
*Atrial Fibrillation*		53 (43.44%)
*Hypercholesterolemia*		61 (50%)
*Diabetes*		29 (23.77%)
***Number of comorbidities***	0	8 (6.56%)
	1	31 (25.41%)
	2+	83 (68.03%)
*Median [q1-Q3]*		2 [1; 3]
***Etiology***	Cardioembolic	62 (51.67%)
	Atherosclerosis	38 (31.67%)
	Other	20 (16.67%)
***Time of the day***		
*Night (20:00, 08:00)*		34 (27.87)
***mRS_pre***		
*Mean (SD)*		1.4 (1.14)
*Median [q1-Q3]*		1 [0; 2]
***NIHSS entrance***		
*Mean (SD)*		17.64 (6.17)
*Median [q1-Q3]*		18 [15; 22]
***Time to CT***		
*Mean (SD)*		121.74 (55.19)
*Median [q1-Q3]*		120 [90; 150]
***Fibrinolysis***		
*Time, minutes*		
*Mean (SD)*		172.25 (55.84)
*Median [q1-Q3]*		170 [150; 200]
***Time to Groin***		
*Mean (SD)*		262.42 (76.79)
*Median [q1-Q3]*		262.5 [210; 312]
***Procedural Time***		
*Mean (SD)*		56.1 (25.01)
*Median [q1-Q3]*		55 [36; 70]

**Table 2:** Univariate analysis for predictors of good outcome at 90 days after endovascular thrombectomy.

Variable		Bad Functional Outcome (n = 66)	Good Functional Outcome (n = 56)	*p*-Value	OR [95% CI]
***Sex***	Female vs. male	44 (66.67)	41 (73.21)	0.4330	1.37 [0.63; 2.99]
***Age, years***	Median [q1-Q3]	84 [82; 87]	82 [81; 84]	**0.0053**	0.87 [0.78; 0.97]
	85+	30 (45.45)	13 (23.21)	**0.0104**	0.36 [0.17; 0.80]
***Side***	Right	35 (53.85)	20 (39.22)	0.1173	-
	Left	30 (46.15)	31 (60.78)		1.81 [0.86; 3.81]
***Circle***	Posterior	2 (3.03)	6 (10.71)	0.1410	3.84 [0.74; 19.85]
***Spoke***		31 (46.97)	14 (25.00)	**0.0122**	0.38 [0.17; 0.82]
***Wake-up Stroke***		10 (15.15)	4 (7.14)	0.1667	0.43 [0.13; 1.46]
***Fibrinolysis***		34 (53.97)	31 (55.36)	0.8793	1.06 [0.51; 2.18]
	Median [q1-Q3]	168.50 [150; 185]	170 [140; 219]	0.8077	1.00 [0.99; 1.01]
***Device***	Direct Aspiration	40 (62.5)	37 (66.07)	0.9024	1.14 [0.52; 2.49]
	Stent retriever	3 (4.69)	2 (3.57)		0.82 [0.12; 5.51]
	“Solumbra”	21 (32.81)	17 (30.36)		-
***N_attempts***	Median [q1-Q3]	1.5 [1; 2]	1 [1; 2]	0.2482	0.84 [0.59; 1.2]
***Rescue***		12 (18.18)	8 (14.29)	0.5624	0.75 [0.28; 1.99]
***Hemorrhage***		30 (45.45)	10 (17.86)	**0.0012**	0.26 [0.11; 0.6]
***Comorbidities***					
*Hypertension*		40 (60.61)	42 (75.00)	0.0915	1.95 [0.89; 4.26]
*Smoke*		5 (7.58)	15 (26.79)	0.0043	4.46 [1.51; 13.23]
*Coronary Artery Disease*		20 (30.30)	18 (32.14)	0.8269	1.09 [0.51; 2.35]
*Atrial Fibrillation*		30 (45.45)	23 (41.07)	0.6265	0.84 [0.41; 1.72]
*Hypercholesterolemia*		27 (40.91)	34 (60.71)	0.0292	2.23 [1.08; 4.62]
*Diabetes*		14 (21.21)	15 (26.79)	0.4711	1.36 [0.59; 3.13]
***Etiology***	Cardioembolic	32 (49.23)	30 (54.55)	0.5643	1.67 [0.55; 5.11]
	Atherosclerosis	20 (30.77)	18 (32.73)		1.74 [0.61; 4.95]
	Other	13 (20.00)	7 (12.73)		
***Time of the day***					
*Night (20:00, 08:00)*		18 (27.27)	16 (28.57)	0.8733	1.07 [0.48; 2.36]
***mRS_pre***	Median [q1-Q3]	1 [1; 2]	1 [0; 2]	0.5700	0.94 [0.69; 1.29]
***NIHSS entrance***	Median [q1-Q3]	20 [17; 22]	17 [9; 22]	**0.0014**	0.88 [0.83; 0.95]
***Time to CT***	Median [q1-Q3]	120 [90; 140]	120 [90; 150]	0.6766	1 [0.99; 1]
***Time to Groin***	Median [q1-Q3]	275 [220; 315]	249 [202.5; 310]	0.1591	1 [0.99; 1]
***Procedural Duration***	Median [q1-Q3]	52 [36; 72]	55 [37.5; 70]	0.7570	1 [0.98; 1.01]

**Table 3:** Multivariable analysis for predictors of good outcome at 90 days after endovascular thrombectomy.

*Multivariable Analysis*	
	OR [95%CI]
*NIHSS at admission*	0.89 [0.82; 0.95]

**Table 4:** Univariate analysis for predictors of good recanalization (TICI ≥ 2b).

Variable		Unsuccessful Recanalization (n = 42)	Successful Recanalization (n = 80)	*p*-Value	OR [95% CI]
***Sex***	Female vs. male	28 (66.67)	57 (71.25)	0.6008	1.24 [0.56; 2.77]
***Age, years***	Median [q1-Q3]	85 [83; 87]	82 [81; 84.5]	**0.0002**	0.88 [0.8; 0.98]
	85+	23 (54.76)	20 (25.00)	**0.0011**	0.28 [0.13; 0.61]
***Side***	Right	20 (48.78)	35 (46.67)	0.8275	-
	Left	21 (51.22)	40 (53.33)		1.09 [0.51; 2.33]
***Circle***	Posterior	2 (4.76)	6 (7.5)	0.7134	1.62 [0.31; 8.41]
***Spoke***		17 (40.48)	28 (35)	0.55142	0.79 [0.37; 1.71]
***Wake-up Stroke***		4 (9.52)	10 (12.5)	0.76931	1.36 [0.4; 4.62]
***Fibrinolysis***		21 (51.22)	44 (56.41)	0.58886	1.23 [0.58; 2.63]
	Median [q1-Q3]	180 [160; 195]	165 [140; 210]	0.4222	1 [0.99; 1.01]
***Device***	Direct Aspiration	24 (60.00)	53 (66.25)	0.5469	1.44 [0.64; 3.24]
	Stent retriever	1 (2.50)	4 (5.00)		2.61 [0.27; 25.65]
	“Solumbra”	15 (37.50)	23 (28.75)		
***N_attempts***	Median [q1-Q3]	2 [1; 2]	1 [1; 2]	0.1171	0.82 [0.58; 1.17]
***Rescue***		11 (26.19)	9 (11.25)	**0.0342**	0.36 [0.13; 0.95]
***Hemorrhage***		17 (40.48)	23 (28.75)	0.1899	0.59 [0.27; 1.3]
***Comorbidities***					
*Hypertension*		30 (71.43)	52 (65)	0.47235	0.74 [0.33; 1.67]
*Smoke*		5 (11.9)	15 (18.75)	0.33188	1.71 [0.57; 5.08]
*Coronary Artery Disease*		10 (23.81)	28 (35)	0.20475	1.72 [0.74; 4.01]
*Atrial Fibrillation*		19 (45.24)	34 (42.5)	0.7719	0.89 [0.42; 1.9]
*Hypercholesterolemia*		16 (38.1)	45 (56.25)	0.05671	2.09 [0.97; 4.48]
*Diabetes*		10 (23.81)	19 (23.75)	0.99414	1 [0.41; 2.4]
***Etiology***	Cardioembolic	22 (52.38)	40 (51.28)	0.99164	1.04 [0.33; 3.23]
	Atherosclerosis	13 (30.95)	25 (32.05)		0.98 [0.34; 2.81]
	Other	7 (16.67)	13 (16.67)		
***Time of the day***					
*Night (20:00, 08:00)*		8 (19.05)	26 (32.50)	0.1154	2.05 [0.83; 5.04]
***mRS_pre***	Median [q1-Q3]	1 [1; 3]	1 [0; 2]	0.1439	0.77 [0.55; 1.07]
***NIHSS entrance***	Median [q1-Q3]	19 [16; 22]	18 [14.5; 22]	0.6213	0.98 [0.92; 1.04]
***Time to CT***	Median [q1-Q3]	120 [90; 150]	120 [90; 145]	0.2807	1 [0.99; 1.01]
***Time to Groin***	Median [q1-Q3]	279.5 [220; 321]	260 [207.5; 305]	0.1968	1 [0.99; 1]
***Procedural Duration***	Median [q1-Q3]	60.5 [45; 80]	50 [32; 70]	**0.0346**	0.99 [0.97; 1]

**Table 5:** Univariate analysis for predictors of good outcome considered as mRS < 3 at 90 days after endovascular thrombectomy.

Variable		Bad Functional Outcome (n = 73)	Good Functional Outcome (n = 49)	*p*-Value	OR [95% CI]
***Sex***	Female vs. male	50 (68.49)	35 (71.43)	0.7295	1.15 [0.52; 2.54]
***Age, years***	Median [q1-Q3]	84 [82; 87]	82 [81; 84]	**0.0067**	0.87 [0.77; 0.98]
	85+	33 (45.21)	10 (20.41)	**0.0049**	0.31 [0.14; 0.72]
***Side***	Right	37 (52.11)	18 (40.00)	0.2030	-
	Left	34 (47.89)	27 (60.00)		1.63 [0.77; 3.48]
***Circle***	Posterior	3 (4.11)	5 (10.2)	0.1825	2.65 [0.60; 11.65]
***Spoke***		33 (45.21)	12 (24.49)	**0.0201**	0.39 [0.18; 0.87]
***Wake-up Stroke***		11 (15.07)	3 (6.12)	0.1286	0.37 [0.1; 1.39]
***Fibrinolysis***		37 (52.86)	28 (57.14)	0.6440	1.19 [0.57; 2.48]
	Median [q1-Q3]	170 [150; 195]	167.5 [140; 210]	0.9736	1 [0.99; 1.01]
***Device***	Direct Aspiration	45 (63.38)	32 (65.31)	0.9766	1.09 [0.49; 2.41]
	Stent retriever	3 (4.23)	2 (4.08)		1.02 [0.15; 6.86]
	“Solumbra”	23 (32.39)	15 (30.61)		-
***N_passages***	Median [q1-Q3]	1 [1; 2]	1 [1; 2]	0.3648	0.86 [0.6; 1.23]
***Rescue***		13 (17.81)	7 (14.29)	0.6064	0.77 [0.28; 2.09]
***Hemorrhage***		32 (43.84)	8 (16.33)	**0.002**	0.25 [0.1; 0.61]
***Comorbidities***					
*Hypertension*		47 (64.38)	35 (71.43)	0.4164	1.38 [0.63; 3.03]
*Smoke*		6 (8.22)	14 (28.57)	**0.0029**	4.47 [1.58; 12.64]
*Coronary Artery Disease*		22 (30.14)	16 (32.65)	0.7686	1.12 [0.52; 2.45]
*Atrial Fibrillation*		33 (45.21)	20 (40.82)	0.6316	0.84 [0.4; 1.74]
*Hypercholesterolemia*		32 (43.84)	29 (59.18)	0.0965	1.86 [0.89; 3.87]
*Diabetes*		16 (21.92)	13 (26.53)	0.5574	1.29 [0.55; 2.99]
***Etiology***	Cardioembolic	36 (50)	26 (54.17)	0.0606	1.70 [0.54; 5.38]
	Atherosclerosis	22 (30.56)	16 (33.33)		1.69 [0.57; 4.97]
	Other	14 (19.44)	6 (12.5)		
***Time of the day***					
*Night (20:00, 08:00)*		21 (28.77)	13 (26.53)	0.7871	0.89 [0.4; 2.01]
***mRS_pre***	Median [q1-Q3]	2 [1; 3]	1 [0; 2]	**0.0109**	0.63 [0.44; 0.89]
***NIHSS entrance***	Median [q1-Q3]	20 [17; 23]	16 [9; 19]	**0.0004**	0.88 [0.83; 0.95]
***Time to CT***	Median [q1-Q3]	120 [90; 140]	120 [90; 150]	0.3635	1 [0.99; 1]
***Time to Groin***	Median [q1-Q3]	275 [225; 315]	245 [190; 310]	0.0962	1 [0.99; 1]
***Procedural Duration***	Median [q1-Q3]	52 [36; 73.5]	55 [40; 70]	0.7835	1 [0.98; 1.01]

## Data Availability

Anonymized data that support the findings of this study are available from the corresponding author, M.C., upon reasonable request.

## References

[1] Strong K., Mathers C., Bonita R. (2007). Preventing stroke: Saving lives around the world. Lancet Neurol..

[2] Béjot Y., Bailly H., Durier J., Giroud M. (2016). Epidemiology of stroke in Europe and trends for the 21st century. Presse Med..

[3] Wolf P.A., Mohr J.P., Choi D.W., Grotta J.C., Weir B., Wolf P.A. (2004). Epidemiology of stroke. Stroke: Pathophysiology, Diagnosis, and Management.

[4] Balami J.S., Sutherland B.A., Edmunds L.D., Grunwald I.Q., Neuhaus A.A., Hadley G., Karbalai H., Metcalf K.A., DeLuca G.C., Buchan A.M. (2015). A systematic review and meta-analysis of randomized controlled trials of endovascular thrombectomy compared with best medical treatment for acute ischemic stroke. Int. J. Stroke.

[5] Goyal M., Menon B.K., van Zwam W.H., Dippel D.W., Mitchell P.J., Demchuk A.M., Dávalos A., Majoie C.B., van der Lugt A., de Miquel M.A. (2016). Endovascular thrombectomy after large-vessel ischaemic stroke: A meta-analysis of individual patient data from five randomised trials. Lancet.

[6] Muir K.W., White P. (2016). HERMES: Messenger for stroke interventional treatment. Lancet.

[7] Kauffmann J., Grün D., Yilmaz U., Wagenpfeil G., Faßbender K., Fousse M., Unger M.M. (2021). Acute stroke treatment and outcome in the oldest old (90 years and older) at a tertiary care medical centre in Germany-a retrospective study showing safety and efficacy in this particular patient population. BMC Geriatr..

[8] Alawieh A., Chatterjee A., Feng W., Porto G., Vargas J., Kellogg R., Turk A.S., Turner R.D., Imran Chaudry M., Spiotta A.M. (2018). Thrombectomy for acute ischemic stroke in the elderly: A ‘real world’ experience. J. Neurointerv. Surg..

[9] Liao G., Zhang Z., Zhang G., Du W., Li C., Liang H. (2021). Efficacy of a Direct Aspiration First-Pass Technique (ADAPT) for Endovascular Treatment in Different Etiologies of Large Vessel Occlusion: Embolism vs. Intracranial Atherosclerotic Stenosis. Front. Neurol..

[10] Delgado Almandoz J.E., Kayan Y., Young M.L., Fease J.L., Scholz J.M., Milner A.M., Hehr T.H., Roohani P., Mulder M., Tarrel R.M. (2016). Comparison of clinical outcomes in patients with acute ischemic strokes treated with mechanical thrombectomy using either Solumbra or ADAPT techniques. J. Neurointerv. Surg..

[11] Guzzardi G., Del Sette B., Stanca C., Galbiati A., Cernigliaro M., Carriero A., Stecco A. (2018). Mechanical Thrombectomy by a Direct Aspiration First Pass Technique (ADAPT) in Ischemic Stroke: Results of Monocentric Study Based on Multimodal CT Patient Selection. Stroke Res. Treat..

[12] Li Z.S., Zhou T.F., Li Q., Guan M., Liu H., Zhu L.F., Wang Z.L., Li T.X., Gao B.L. (2021). Endovascular Management of Intracranial Atherosclerosis-Related Large Vessel Occlusion with the A Direct Aspiration First-Pass Thrombectomy Compared With Solumbra Technique. Front. Neurol..

[13] Kurre W., Aguilar-Pérez M., Martinez-Moreno R., Schmid E., Bäzner H., Henkes H. (2017). Stent Retriever Thrombectomy of Small Caliber Intracranial Vessels Using pREset LITE: Safety and Efficacy. Clin. Neuroradiol..

[14] Lehnen N.C., Paech D., Zülow S., Bode F.J., Petzold G.C., Radbruch A., Dorn F. (2022). First Experience with the Nimbus Stentretriever: A Novel Device to Handle Fibrin-rich Clots. Clin. Neuroradiol..

[15] Borggrefe J., Goertz L., Abdullayev N., Hokamp N.G., Kowoll C.M., Onur Ö., Kabbasch C., Schlamann M. (2021). Mechanical Thrombectomy with the Novel NeVa M1 Stent Retriever: Do the Drop Zones Represent a Risk or a Benefit?. World Neurosurg..

[16] Amireh A.O., Kuybu O., Adeeb N., Kelley R.E., Javalkar V., Cuellar H., Sharma P. (2021). Utilization of the large-bore Penumbra JET 7 reperfusion catheter in thrombectomy for acute ischemic stroke: A single-center experience. Interv. Neuroradiol..

[17] Essibayi M.A., Brinjikji W. (2022). Efficacy and safety of SOFIA aspiration catheter for mechanical thrombectomy via ADAPT and Solumbra echniques in acute ischemic stroke: A systematic review and meta-analysis. Interv. Neuroradiol..

[18] Sharobeam A., Cordato D.J., Manning N., Cheung A., Wenderoth J., Cappelen-Smith C. (2019). Functional Outcomes at 90 Days in Octogenarians Undergoing Thrombectomy for Acute Ischemic Stroke: A Prospective Cohort Study and Meta-Analysis. Front Neurol..

[19] Sussman E.S., Martin B., Mlynash M., Marks M.P., Marcellus D., Albers G., Lansberg M., Dodd R., Do H.M., Heit J.J. (2020). Thrombectomy for acute ischemic stroke in nonagenarians compared with octogenarians. J. Neurointerv Surg..

[20] Groot A.E., Treurniet K.M., Jansen I.G.H., Lingsma H.F., Hinsenveld W., van de Graaf R.A., Roozenbeek B., Willems H.C., Schonewille W.J., Marquering H.A. (2020). Endovascular treatment in older adults with acute ischemic stroke in the MR CLEAN Registry. Neurology.

[21] Botelho A., Rios J., Fidalgo A.P., Ferreira E., Nzwalo H. (2022). Organizational Factors Determining Access to Reperfusion Therapies in Ischemic Stroke-Systematic Literature Review. Int. J. Environ. Res. Public Health.

[22] Saver J.L. (2006). Time is brain--quantified. Stroke.

